# The emerging neuroprotective roles of exerkines in Alzheimer’s disease

**DOI:** 10.3389/fnagi.2022.965190

**Published:** 2022-08-31

**Authors:** Tayna Rody, Julia A. De Amorim, Fernanda G. De Felice

**Affiliations:** ^1^Institute of Medical Biochemistry Leopoldo de Meis, Federal University of Rio de Janeiro, Rio de Janeiro, Brazil; ^2^Centre for Neuroscience Studies, Department of Biomedical and Molecular Sciences, Queen’s University, Kingston, ON, Canada; ^3^Department of Psychiatry, Queen’s University, Kingston, ON, Canada; ^4^D’Or Institute for Research and Education, Rio de Janeiro, Brazil

**Keywords:** Alzheimer’s disease (AD), physical exercise, exerkines, irisin, cognition, neuroprotection

## Abstract

Despite the extensive knowledge of the beneficial effects of physical exercise, a sedentary lifestyle is still a predominant harm in our society. Sedentarism is one of the major modifiable risk factors for metabolic diseases such as diabetes mellitus, obesity and neurological disorders, including Alzheimer’s disease (AD)–characterized by synaptic failure, amyloid protein deposition and memory loss. Physical exercise promotes neuroprotective effects through molecules released in circulation and mediates the physiological crosstalk between the periphery and the brain. This literature review summarizes the current understanding of the roles of exerkines, molecules released during physical exercise, as systemic and central factors that mediate the beneficial effects of physical exercise on cognition. We highlight the neuroprotective role of irisin—a myokine released from the proteolytic cleavage of fibronectin type III domain-containing protein 5 (FNDC5) transmembrane protein. Lastly, we review evidence pointing to physical exercise as a potential preventative and interventional strategy against cognitive decline in AD.

## Introduction

A challenging lifestyle modification that is critically needed for modern societies consists in decreasing sedentary habits (Pratt et al., 2014; Trost et al., 2014). Unlike our ancestors who lived as nomads hunting and acquiring their food, our moving from the countryside to the city as modern society caused changes in the human diet and propitiated sedentary behavior. The progressive and continuous change over the decades has been increasingly associated with alterations in systemic and brain healthy aging, favoring the development of diseases that are linked to an increased risk of dementia (De Felice et al., 2022). In this context, a recent study observed that the prevalence of dementia in the indigenous population of Tsimane and Moseten Amerindian of the Bolivian Amazon, who live a subsistence lifestyle, hunting, gathering food from the forest, and fishing, was one of the lowest compared with the majority population of the world. These observations evidence that a physically active lifestyle may be a critical factor in establishing improved body and brain health (Gatz et al., 2022).

Indeed, the association between a healthy lifestyle and physical activity is not recent. Galen’s (150 or 130-201 AC) studies were among the first to exemplify the close relationship between medicine and sports. In Ancient Greece, athletes were prepared physically and physiologically by trainers. In addition, Hippocrates, known as the “Father of Medicine,” was a pioneer in prescribing exercise and diet to patients (Masters, 1976; Tipton, 2015).

Despite growing scientific evidence of the beneficial effects of physical exercise, according to the World Health Organization (WHO), one in four adults doesn’t exercise the minimum stipulated by global recommendations. It is estimated that four to five million deaths per year could be avoided if the global population was more physically active (World Health Organization [WHO], 2022). While many factors can contribute to the development and progression of Alzheimer’s disease (AD), physical inactivity has been increasingly considered to be important in favoring the high incidence of the AD cases (Blair, 2009; Pratt et al., 2014; Reiman, 2014). Physical inactivity favors the surge of several cardiovascular and metabolic diseases, such as hypertension, diabetes, and obesity, which have been associated to increase the risk of developing AD (MacIntosh et al., 2010; Felice, 2013; Clarke et al., 2018). Conversely, a well-established factor is that interventions in active lifestyles, such as exercising regularly, could reduce cognitive decline and improve overall health during aging.

Herein, we review the molecular mechanisms underlying the neuroprotective actions of exercise that make it a potential preventative and interventional strategy against cognitive decline in AD. We discuss the role of exerkines, signaling molecules released by different tissues in response to acute and/or chronic exercise that exerts their effects through endocrine, paracrine and/or autocrine manners – as systemic and central mediators that contribute to the beneficial effects of exercise in cognition, including the fibronectin type III domain-containing protein 5 (FNDC5)/irisin, cathepsin B (CTSB), 3-Hydroxybutyrate (3OHB), lactate, interleukin-6 (IL-6), and brain-derived neurotrophic factor (BDNF). We highlight the role of FNDC5/irisin as a myokine associated with neuroprotective effects in AD. Overall, we present mounting evidence that helps to explain why physical activity is beneficial to healthy brain aging.

## Alzheimer’s disease pathophysiology

Alzheimer’s disease is a progressive neurodegenerative disease and is the main form of dementia in the world. Changes in mood, personality, hallucinations, and memory loss are the most recognized symptoms of AD (Masters et al., 2015). AD is a highly prevalent disease associated with aging. It is estimated to have a rise in prevalence in the following decades (Association, 2013; Alzheimer’s Dement, 2020). Age is the central risk factor for AD, and growing evidence suggests that dietary and lifestyle changes are leading to a faster and more significant increase in the incidence of AD. In addition, common diseases in modern life, such as diabetes mellitus, obesity, and depression, have been identified as strong risk factors for AD establishment at older ages (Daviglus et al., 2011; Selkoe, 2012; Ledo et al., 2013, 2016; De Felice et al., 2014; Lloret et al., 2019; Jayaraj et al., 2020).

The main hallmarks of AD pathology comprise amyloid-beta (Aβ) plaque accumulation, Aβ oligomer (AβO) formation and tangle formation formed by abnormally hyperphosphorylated tau, known as neurofibrillary tangles (NFTs) (Masters et al., 2015). These histopathological alterations are associated with the severity of the disease. As AD progresses, amyloid plaques and NFTs aggregates accumulate in many brain regions, including areas critical for cognition. Neurodegeneration, neuroinflammation, and synaptic loss are key features of the disease (Masters et al., 2015; Alzheimer’s Dement, 2020).

Considering the complexity of the AD spectrum and the lack of efficient pharmacological therapies, the interest in non-pharmacological strategies is being increasingly investigated. Due to this presently scenario, physical exercise is displayed as a potential adjunctive therapy. Physical exercise is an attractive approach that conducts a beneficial effect on mental and physical health. Physical exercise can be defined as a structured, repetitive, and planned activity that aims to improve or maintain physical fitness (Caspersen et al., 1985; Masters et al., 2015).

## Neurological benefits of physical exercise

Growing evidence from human and mouse studies shows that exercise can modulate multiple mechanisms associated with brain and periphery functions. Amongst these mechanisms, we will highlight the effects of exercise on altered processes in the AD brain. Exercise stimulates hippocampal volume, neurogenesis, vascular function, and growth factors cascades. Also, physical exercise is associated with decreases in hippocampal atrophy, neuroinflammation, and amyloid plaque load (Ahlskog et al., 2011; Erickson et al., 2012; Liu et al., 2012; Braskie et al., 2014; Yang et al., 2014; Tarumi et al., 2019).

The neuroprotective processes stimulated by physical exercise have been well-investigated in mouse models of AD and humans. From an anatomical standpoint, the hippocampal volume shrinks 1–2% yearly in older adults without dementia (Pratt et al., 2014) and decreased hippocampal volume is associated with cognitive impairment (Armstrong et al., 2020). Aerobic exercise has been shown to prevent brain volume reductions associated with oldness (Colcombe and Kramer, 2003). Another study showed that a 1-year aerobic exercise intervention can reverse hippocampal volume loss in late adulthood, enhancing volume and cognition (Erickson et al., 2011). Decreased brain volume is associated with AD progression (Cha et al., 2004; Fotenos et al., 2005, 2008). Several studies have focused on evaluating the effects of exercise on brain volume, especially in the hippocampus (Firth et al., 2018), a brain area affected in AD. An analysis performed during 6 months with randomized older women suffering from probable mild cognitive impairments showed that aerobic training significantly increased hippocampal volume (Ten Brinke et al., 2014). Tarumi and colleagues also examined aerobic training in patients diagnosed with amnestic mild cognitive impairment for 12 months. In amyloid-positive patients, aerobic exercise reduced hippocampal atrophy (Tarumi et al., 2019). Consistently, exercise influences hippocampal functional connectivity strengthening and rewiring neuronal networking (Ten Brinke et al., 2014; Voss et al., 2019).

The neuroprotective processes stimulated by physical exercise have been well-investigated in mouse models. Studies report that exercise attenuates the cognitive decline in behavioral tests, such as spatial learning and memory, contextual memory, passive avoidance, novel object recognition (NOR), and recognition memory (Vaynman et al., 2004; Van Praag et al., 2005; Radak et al., 2006; Schweitzer et al., 2006; O’Callaghan et al., 2007; Ma et al., 2017; Lourenco et al., 2019; Voss et al., 2019). Furthermore, a central question in studying the effects of physical exercise on the brain concerns the best regimen that could improve the benefits. A comparative study evaluated the impact of 1-month and 2-month voluntary running in 3-month-old TgCRND8 mice (Maliszewska-Cyna et al., 2016). While both protocols improved memory, only the 2-month intervention protocol showed hippocampal neurogenesis and reduced plaque load. These data indicate that extended intervention would be preferable to favor neuroprotective pathways (Maliszewska-Cyna et al., 2016).

Physical exercise has been shown to stimulate neurogenesis, a process that is inhibited in animal models of AD. In APP/PS1 [mice harboring the Swedish amyloid precursor protein (APP) mutation and deletion of the exon 9 of presenilin-1] transgenic mice, treadmill exercise enhanced learning and memory possibly by adjusting the amyloid precursor protein (APP) proteolytic pathway. However more studies are needed to better elucidate how newborn cells act. Notably, after 12 weeks, treadmill exercise significantly rose the number of proliferating cells in both wild-type mice and APP/PS1 transgenic mice (Yu et al., 2021). Interestingly, Choi and co-workers showed that combining adult hippocampal neurogenesis induced genetically and pharmacologically together with BDNF mimic exercise ameliorated the cognitive defect in AD transgenic 5xFAD. Additionally, this protocol reduced Aβ load and elevated levels of BDNF, IL-6 and FNDC5 (Choi et al., 2018). Also, exercise-induced hippocampal neurogenesis is associated with the stimulation of mechanisms linked to synaptic plasticity, learning, and memory (Van Praag et al., 1999a; Trejo et al., 2001; Fabel et al., 2003; Klempin et al., 2013). In mice, voluntary running increased neurogenesis, inducing changes in cell proliferation. Neurogenesis is more evident in the dentate gyrus, where long-term potentiation (LTP) and short-term potentiation are increased (Blumenthal et al., 1991; Van Praag et al., 1999a,b; Chatzi et al., 2019). Indeed, the improvement in hippocampal neurogenesis was shown to contribute to synaptic plasticity (Van Praag et al., 2005; Ma et al., 2017), promoting synapse formation, strengthening, and function (Cotman et al., 2007).

Innumerous studies evaluated the main hallmarks involved in the pathophysiology of AD. Tapias-Rojas and colleagues showed that voluntary exercise decreased Aβ burden, Thioflavin-S-positive plaques and Aβ oligomers in the APPswe/PS1dE9 mice, a transgenic mouse model of AD (Tapia-Rojas et al., 2016). In addition, APPswe/PS1dE9 mice showed decreases in the levels of tau phosphorylation (Tapia-Rojas et al., 2016). Likewise, treadmill exercise decreased soluble Aβ1-42 levels and the deposits of Aβ in the hippocampus and cerebral cortex of 3xTg-AD mice, a triple transgenic mouse model of AD (Cho et al., 2015). Thomas et al. (2020) further showed that exercise training decreased amyloid plaque load in Tg2576 mice. Together these findings demonstrate that exercise may exert its beneficial actions in the brain by modulating disease-associated factors.

Alzheimer’s disease development is related to metabolic, hypertension, and cardiovascular diseases (MacIntosh et al., 2010; Felice, 2013; Clarke et al., 2018). Exercise reduces peripheral risk factors by inducing growth factors cascades (Trejo et al., 2001; Fabel et al., 2003; Petersen and Pedersen, 2005). A common mechanism underlying the effects of exercise in peripheral and brain disorders might be associated with inflammation, which can impair growth factor signaling both systematically and centrally. Studies demonstrated that physical exercise reduces the levels of GFAP + cells and lower microglial activation in transgenic APPswe/PS1Δ*E9* mice (Tapia-Rojas et al., 2016; Zhang et al., 2018). In the Tg2576 mouse model, 3 weeks of voluntary wheel running decreased the levels of pro-inflammatory interleukin-1β (IL-1β) and tumor necrosis factor α (TNF-α) (Nichol et al., 2008; Parachikova et al., 2008). The anti-inflammatory actions of exercise are thought to improve the brain redox status, thereby ameliorating the pathophysiological hallmarks of AD (García-Mesa et al., 2011; Garciá-Mesa et al., 2015). Therefore, current evidence indicates that during physical activity, the release of systemic and central molecules may contribute to the beneficial effects of exercise on the central nervous system (CNS) (Cotman et al., 2007; Safdar and Tarnopolsky, 2018).

## The neuroprotective potential of exerkines

Recent studies have been trying to identify and better comprehend the possible mechanisms underlying the positive effects of exercise and the potential to treat diseases. Currently, exerkines are under discussion as they might be key components responsible for orchestrating the beneficial effects of exercise (Gupta et al., 2021; Chow et al., 2022).

**FIGURE 1:**
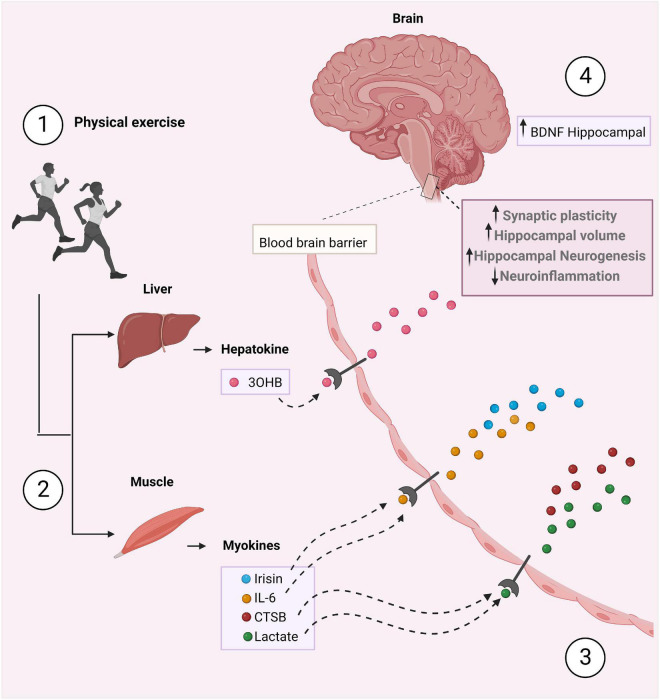
The crosstalk between the periphery and the brain stimulated by physical exercise. (1) During physical exercise, peripheral tissues secrete exerkines, such as irisin, IL-6, CTSB, 3OHB, and lactate which mediate the beneficial effects of exercise (2). These exerkines cross the BBB (3), increase BDNF levels, hippocampal volume and neurogenesis, and decrease neuroinflammation (4). As a result, improved synaptic plasticity and memory occurs. Figure was created with BioRender.com.

A multitude of organs, cells and tissues release those factors, including skeletal muscle (myokines), white adipose tissue (adipokines), liver (hepatokines), and neurons (neurokines) (Chow et al., 2022). Understanding the role of exerkines in the physiological and biological response to exercise is essential to better understand their possible role in treating diseases. However, endless questions remain poorly understood. Therefore, growing studies are focusing on the investigation of exerkines and their mechanisms of action in multiple organ systems. In this following section, we aim to elucidate the mechanisms by which exerkines have been described to mediate the neuroprotective effects of exercise. Herein, we provide information about six exerkines: IL-6, CTSB, 3OHB, lactate, BDNF, and FNDC5/irisin (Figure 1) (Supplementary Table 1).

### IL-6

Primarily, interleukin-6 (IL-6) was recognized for its pro-inflammatory effects. Specifically, in AD patients, elevated IL-6 levels in the brain and the blood are associated with the severity of dementia (Huell et al., 1995; Kálmán et al., 1997). Recent data provided by Lyra e Silva et al. (2021) demonstrated that brain IL-6 signaling is upregulated and mediates memory impairment in AD mouse models. Additionally, elevated IL-6 adhesive to senile plaque inclusion levels was found in immunohistochemistry of AD patients (Licastro et al., 2003; Erickson and Banks, 2011).

Despite having a pro-inflammatory role, IL-6 has been shown to be involved differently in physiological and pathological conditions. A study demonstrated that human skeletal muscle contraction releases considerable amounts of IL-6 into the bloodstream during physical exercise (Steensberg et al., 2000). A notion emerged that IL-6 released by skeletal muscle could have a role in the metabolic adaptations promoted by exercise (Gleeson, 2000; Keller et al., 2001; Steensberg et al., 2001). Indeed, IL-6 has been assigned the role of energy sensor (Pedersen et al., 2004; Ruderman et al., 2006; Hoene and Weigert, 2007; Pedersen, 2012), allowing glycemic control by stimulating the oxidation of glucose (Carey et al., 2006) and fatty acid oxidation (Van Hall et al., 2003; Petersen et al., 2005).

From a CSN perspective, studies found that exercise modulates the inflammatory profile of cytokines in Tg2576 AD’S transgenic mice (Parachikova et al., 2008). Furthermore, another study evaluated increases in the concentration of pro-inflammatory cytokines in the plasma and cerebrospinal fluid (CSF) of 198 AD patients before and after a 16-week intervention protocol and showed increased IL-6 plasma levels in the exercised group compared to controls (Jensen et al., 2019).

### Cathepsin B

Cathepsin B (CTSB), a cysteine protease, is an exercise-induced muscle secretory factor linked to hippocampal functions (Pedersen, 2019). Moon et al. (2016) demonstrated that CTSB crosses the blood-brain barrier in mice, mediating a central increase in BDNF and doublecortin (DCX). Both proteins are related to neuroprotective effects, especially in hippocampal neurogenesis and neuronal migration. Moreover, 14 or 30 days of running increased both skeletal, and muscular expression of the CTSB gene and CTSB protein levels in mice plasma. Interestingly, exercise could not enhance neurogenesis and improve spatial memory in CTSB knockout mice. CTSB protein levels in plasma were also enhanced in rhesus monkeys and humans subjected to 4 months of treadmill training. A complex figure (CF) drawing recall test was performed, and those with higher plasma levels of CTBS obtained higher test scores (Moon et al., 2016). Additionally, in hippocampal progenitor cells, CTSB administration was able to increase the expression of BDNF mRNA and BDNF protein levels and DCX protein levels (Moon et al., 2016).

In the context of AD, studies with hAPP mice have shown that CTSB has an anti-amyloidogenic action, decreasing Aβ plaque deposition in mice hippocampus and cortex. CTSB can act by proteolytic cleavage, originating alternatives Aβ peptides with less amyloidogenic tendencies (Mueller-Steiner et al., 2006). In CTSB knockout mice (CTBS−/−), Aβ levels and amyloid plaque deposition were increased when compared to wild type mice (CTSB+/+) (Mueller-Steiner et al., 2006). In accordance with these findings, lower levels of cystatin-C (CysC), an CTSB inhibitor, were associated with reduced soluble Aβ levels and plaque load in hAPP-J20 mice (Sun et al., 2008). Controversially, other CTSB inhibitors such as CA074Me and E64d were able to reduce amyloid plaque deposition in the brain of London APP mice expressing the wild type β-secretase site of APP (Hook et al., 2008). These two inhibitors were also effective in diminishing brain levels of Aβ40 and Aβ42 and improving spatial memory capability in the Morris water maze test (MWM) (Hook et al., 2008). Altogether, these findings suggest a positive correlation between cathepsin B and the benefits of exercise in hippocampal function, as well as a defining role, although not well-comprehended yet, in the modulation of amyloid plaques.

### 3-Hydroxybutyrate

Exerkines are not exclusively proteins. Other types of molecules that participate in regulating metabolic energy expenditure are also stimulated by vigorous exercise and other physiological conditions that reduce plasma glucose levels. Posterior to the mobilization and beta-oxidation of fatty acids, there is a hepatic production of ketone bodies, mainly 3-Hydroxybutyrate (3OHB) (Marosi et al., 2016). 3OHB can cross the blood brain barrier (BBB) and act as an energy source for neurons when extracellular glucose levels in the brain are insufficient to maintain neuronal function (Pedersen, 2019; Valenzuela et al., 2020). Marosi et al. demonstrated that 3OHB could activate the BDNF gene promoter IV in cerebral cortical neurons through a signaling pathway that involves transcription factor nuclear kappa B (NF-κB) and histone acetyltransferase p300. All three components seem to be needed to observe the positive effects since NF-κB and p300 inhibitors were capable of suppressing 3OHB induced BDNF gene expression. This expression is also dependent on extracellular glucose levels. In a low glucose concentration (1 mM), 3OHB stimulated BDNF expression, contrary to high glucose concentrations (10 mM), where the results were not perceived. In addition, 3OHB circulating levels correlated to hippocampal BDNF levels in mice subjected to 6 weeks of voluntary exercise and were higher than the same parameters in sedentary mice (Marosi et al., 2016).

In cultured hippocampal neurons from rat embryos that were exposed to 5 μM of Aβ1–42 for 14 h, treatment with 4 mM of D-β-Hydroxybutyrate was capable of ameliorating Aβ1–42 toxicity, doubling the number of surviving cells and increasing cell size and neurite outgrowth, when compared to neurons that were exposed only to Aβ1–42 (Kashiwaya et al., 2000). Interestingly, a study found that AD patients have significantly lower levels of β-Hydroxybutyrate (BHB) in their brain tissue and red blood cells, when compared to control groups (Shippy et al., 2020). The same study demonstrated that 5XFAD mice treated with BHB enriched water had a significantly lower percentage area and volume covered by amyloid-β plaques, in comparison to 5XFAD mice that received untreated water (Shippy et al., 2020). With respect to behavioral and cognitive performances, treatment with ketone bodies BHB and acetoacetate (ACA) improved APP mice response to morris water maze (MWM) and NOR tests (Yin et al., 2016). APP + ketones mice performance in the MWM was closer to wild type mice than APP untreated mice. In the NOR test, APP + ketones mice spent more time exploring the novel objects, when compared to APP untreated mice (Yin et al., 2016).

These findings suggest that 3OHB could also be responsible for BDNF increase in physical exercise. Since BDNF plays a critical role in regulating hippocampal-dependent memory, 3OHB could contribute to its neuroprotective properties in neurodegenerative diseases, such as AD and other forms of dementia.

### Lactate

Lactate is the central molecule produced by anaerobic metabolism in the human body (Goodwin et al., 2007). It is highly stimulated by red blood cells since they don’t have mitochondria to perform oxidative phosphorylation and by myocytes in intense exercise, when oxygen levels are too low to maintain aerobic metabolism (Stallknecht et al., 1998). Most of the lactate produced is released in the circulation, where some tissues can convert it back to pyruvate and use it to form glucose, such as the liver and the heart. Lactate can also cross the BBB and be used as an energy source in the brain, similar to ketone bodies (Valenzuela et al., 2020). According to recent studies, lactate also promotes BDNF expression in the hippocampus by inducing the activation of Sirtuin1 deacetylase (SIRT1). SIRT1 interacts with transcriptional coactivator Peroxisome proliferator-activated receptor gamma coactivator 1-alpha (PGC1-α), which regulates the secretion of myokine FNDC5/irisin, already known to elevate BNDF expression (El Hayek et al., 2019). To examine whether this increase in BDNF was linked to improved learning and spatial memory formation, Hayek and colleagues performed the MWM test in wild-type control and lactate receiving mice (180 mg/kg). The second group had a better performance in the trial, showing that increased BDNF plays a role in hippocampal learning and memory formation (El Hayek et al., 2019). Together, these data support the importance of exercise-induced lactate production, which culminates with higher hippocampal BDNF expression. Since the hippocampus is essential to memory function, and memory loss is a critical symptom of AD, lactate could play an important role in its prevention and future treatments.

### Brain-derived neurotrophic factor

The exerkines cited above seem to act through a similar manner. They are secreted into circulation peripherally, cross the BBB and stimulate the production and expression of BDNF in the CNS. BDNF is part of the neurotrophin family, meaning it plays an essential role in neuronal differentiation and survival, synaptic integrity, brain plasticity and memory function (Piepmeier and Etnier, 2015). BDNF is highly expressed in the hippocampus, a brain area that is closely related to learning and memory formation. Patients diagnosed with AD, a disease marked by memory deficits, have lower BDNF levels concentrations in blood and in the CNS (Valenzuela et al., 2020).

BDNF production is stimulated by acute physical exercise, both in healthy adults and in AD patients (Valenzuela et al., 2020). Since BDNF has also been shown to promote the growth and proliferation of hippocampus cells, physical exercise could indirectly mediate these effects. Aerobic exercise training for three months could increase hippocampal volume in human trials (Pedersen, 2019). Considering the decrease in brain volume in AD patients, Erickson et al. suggested a correlation between this decrease, peripheral measures of BDNF (pBDNF) and memory in older adults. Individuals were tested with spatial memory tasks. The older participants, who had lower concentrations of pBDNF, had worse performance on the test and a smaller hippocampus when compared to the younger group. However, low levels of pBDNF were associated with worse test performance and a smaller hippocampus, independently of the participants’ age (Erickson et al., 2010). These results converge with the extensive literature surrounding the importance of exercise and BDNF on cognitive performance.

### The role of FNDC5/irisin in Alzheimer’s disease

FNDC5/irisin is another recently described exerkine that was shown by our group to have neuroprotective roles in AD models (Lourenco et al., 2019). FNDC5 is composed of a signal peptide at the N-terminus composed of 29 amino acids (aa), a fibronectin III (FN III) domain with 94 aa, an undefined fragment with 28 aa, a transmembrane domain with 19 aa, and a hydrophobic domain at the C-terminus having 39 aa and the gene FNDC5 is located on the p arm of the human chromosome (Boström et al., 2012). FNDC5 has a crystallographic structure that indicates a dimeric conformation with great potential to be a membrane receptor (Schumacher et al., 2013). Although the enzymes involved in the cleavage of FNDC5 are not elucidated, irisin is known to be the secreted form (Boström et al., 2012).

Irisin is an N-terminal fragment composed of 112 aa residues, polypeptide hormone produced by skeletal muscle and released into the bloodstream during physical activity as a cleavage product of the FNDC5 (Boström et al., 2012). Schumacher et al. (2013) visualized by crystallography the conformation of monomeric and dimeric form irisin as an antiparallel B-plated sheet. Mouse, rat and human irisin are 100% identical (Schumacher et al., 2013). Irisin is predicted to have a 12 kDa molecular weight. However, it is important to notice irisin undergoes post-translational modifications, such as as glycosylation, which modifies the final molecular weight observed in western blotting (Albrecht et al., 2020). Therefore, more studies are needed to investigate the glycosylation process of FNDC5/irisin.

Firstly, from a physiological standpoint, FNDC5/irisin was identified to regulate metabolism. Through the transcriptional PPARγ (peroxisome proliferator-activated receptor gamma) coactivator 1α (PGC1-α)/FNDC5 pathway, irisin participates in energy metabolism, activating thermogenic functions in adipose tissue, mitochondrial biogenesis, and in the programming of genes, such as uncoupling protein 1 (UCP-1) and cell death inducing DFFA like effector A (CIDEA) (Boström et al., 2012). Irisin also participates in bone-strengthening *via* the integrin αVβ5 in fat and bone (Kim et al., 2018).

Selectively, exercise induces hippocampal FNDC5, and BDNF expression in a mechanism dependent on PPARγ coactivator 1α (PGC-1α) (Wrann et al., 2013). As well as a knockdown of FNDC5 reduced the central BDNF expression (Severinsen and Pedersen, 2020). These findings suggest that FNDC5 may have a neuromodulatory role, mediating the beneficial effects of exercise on brain functions and cognition. Since the hippocampus is a brain region involved in learning and memory, irisin could be related to AD. Our group demonstrated reduced levels of FNDC5/irisin in the brain of AD patients and AD mouse models (Lourenco et al., 2019). Interestingly, boosting irisin levels using adenoviral vectors or recombinant irisin protected against memory impairment in AD mouse models. Moreover, blockade of either peripheral or brain irisin, using anti-FNDC5/irisin neutralizing antibody, prevented the neuroprotective effects of the physical exercise on memory and synaptic plasticity, indicating that irisin mediates the neuroprotective actions of exercise in the brain of AD mouse models (Lourenco et al., 2019). Irisin levels were further found to be positively correlated with cognitive performance and BDNF levels and inversely correlated with Aβ levels in humans (Lourenco et al., 2020).

Although the mechanisms underlying the actions of the FNDC5/irisin are not totally elucidated, our group demonstrated that FNDC5/irisin stimulated the cyclic Adenosine Monophosphate/protein kinase B/cAMP response element-binding protein (cAMP/PKA/CREB) pathway in mice and human brain slices (Wrann et al., 2013; Lourenco et al., 2019). Of relevance, PGC-1α stimulated by FNDC5/irisin indicates that irisin is an important myokine that participates in the communication between the muscle and the brain (Wrann et al., 2013; Lourenco et al., 2019; de Freitas et al., 2020). Integrin αVβ5 was suggested to be a receptor for irisin. Kim and collaborators identified that irisin promotes bone strengthening *via* the integrin αVβ5 in fat and bone (Kim et al., 2018). Further, Yu et al. (2019) described that irisin concentrations decrease upon rat cardiomyocytes to an inhibitor of disintegrin and metalloproteinase (ADAM) family members, thereby suggesting a possible protease candidate for FNDC5 processing. Recently, an elegant study showed that irisin post-treatment ameliorated neuroinflammation and neuronal apoptosis underlying mechanism involving integrin αVβ5/AMPK pathway (Wang et al., 2022). Together, findings suggest irisin as an important myokine that participates in the communication between the muscle and the brain. Additional studies are warranted to better elucidate the role of irisin in different aspects of physiology and pathology.

On the other hand, although the discoveries about myokine irisin are promising in the peripheral and central fields, many questions have to be resolved. The meta-analysis aimed to identify the long-term effect of physical activity on blood levels and detailed that among 33 studies, the level increased into circulation 23x and decreased 10x (Jandova et al., 2021). The meta-analysis reviewed that physical exercise increases irisin blood levels. However, the long-term effect depends on the type of physical exercise. Overall results so far are still limited and more research on the effects of different types of exercise and exercise protocols on peripheral and central levels of irisin will importantly contribute to the understanding of the roles of irisin in health and disease (Albrecht et al., 2020; Jandova et al., 2021).

In addition, it is important to note the current limitations in detecting irisin. The molecular weight of FNDC5 and irisin appears to be different in tissues due to the glycosylation, and different bands may appear in the western blottings. Many studies detected the molecular weight range from 22 to 30 kDa of the FNDC5 in different species (Roca-Rivada et al., 2013; Brenmoeh et al., 2014; Löffler et al., 2015; Zügel et al., 2016). Our group confirmed four immune-reactive bands of FNDC5 in mouse samples in the range between 27 and 75 kDa by mass spectrometry. Furthermore, Albrecht et al. (2015) detected specific FNDC5 bands by western blotting in murine, bovine, and human muscle, but not in humans. Also, the reliability of irisin levels measured with commercial enzyme-linked immunosorbent assay (ELISA) has been posed. Therefore, the current gold standard protocol for irisin quantification and detection is mass spectrometry (Albrecht et al., 2020).

## Exercise as a non-pharmacological therapeutic approach for Alzheimer’s disease

Alzheimer’s disease is a progressive, complex, multifactorial neurodegenerative disease that remains with no cure. Despite scientific advances in understanding the pathophysiology of AD, most of the results obtained in clinical trials are disappointing. There is an urgent need to develop strategies aimed at preventing the progression of the disease and at reversing cognitive impairment in AD patients.

Over the last decades, few treatments have been approved (Tariot et al., 2004; Noetzli and Eap, 2013; Parsons et al., 2013). The first two classes of medications approved for patient use, cholinesterase inhibitors and N-methyl-D-aspartate (NMDA) receptor antagonists, are used in symptomatic patients that have already developed moderate to severe AD and are limited to alleviating symptoms but don’t stop the progression of the disease. Recently, second-generation therapies became more popular, targeting disease-modifying factors such as Aβ and tau, the two main disease hallmarks responsible for AD progression (Masters et al., 2015). Classes of antibodies aimed at targeting Aβ were highly warranted in the field (Farlow et al., 2012; Sevigny et al., 2016). However, their clinical efficiency is still a controversy (Fillit and Green, 2021; Knopman et al., 2021).

The development of new therapeutics should encompass the complex nature of AD pathology and must be less aggressive and invasive, probably depending on the identification of new molecular mechanisms. Therefore, the next generation of therapy should be able to interfere with the disease at the early stages before symptoms have appeared. Combining multiple healthy lifestyle factors seem to be a promising strategy. As we delineate in this review, exercise could be a critical non-invasive intervention. U.S. Pointer is developing a multicenter randomized clinical trial to protect brain health through lifestyle intervention, aiming to reduce the risk of cognitive defects. The purpose is to test the effectiveness of lifestyle changes (NCT03688126) in protecting brain health.

It is important to take into consideration that strategies including physical exercise as a non-pharmacological treatment in AD are challenging, as a long-term commitment would be needed, and it can be difficult to engage individuals with mild cognitive impairment or AD in this type of therapy. Another challenge consists in determining the ideal type of exercise, i.e., aerobic exercise, endurance, that would be preferable to increase the levels of exerkines in humans. Total intervention period, minutes per week, dementia symptoms stage, and patient age are amongst many of the variables that need to be considered to develop the best treatment protocol.

Although physical exercise is an essential complementary treatment, AD affects mainly older populations that commonly suffer from mobility issues, making it harder for these patients to exercise. Interestingly, a Brazilian group implemented a virtual reality-based physical exercise with exergames in institutionalized older adults. Virtual simulation showed mental and physical health benefits, especially in short-term memory and mobility (Monteiro-Junior et al., 2017). This experiment is limited, and further studies should better indicate the potential beneficial effects of exergames.

In conclusion, exerkines are a group of promising molecules that may underlie the beneficial effects of exercise in individuals with AD (Chow et al., 2022). However, more studies are needed to investigate the signaling pathways of exerkines, providing a deeper understanding of neuroprotective effects of physical exercise. Our recent findings indicate that increased FNDC5/irisin signaling, whether through physical exercise or pharmacologically, aimed to mimetize the effects of exercise, may benefit AD patients (Lourenco et al., 2019). In addition, a recent interesting study suggested that extracellular vesicles (EVs) could orchestrate the multisystem benefits promoted by exercise, mediating the communication between tissues, and resulting in a metabolic improvement (Whitham et al., 2018). Future studies from this exciting field are highly warranted to improve our comprehension of how physical exercise is capable to exert its neuroprotective actions in the CNS.

## Author contributions

FD conceived the idea and edited the manuscript for review. TR and JD contributed to the idea, delineated and wrote the manuscript. All authors approved the submitted version.
