# Supplementary material for: The emerging neuroprotective roles of exerkines in Alzheimer’s disease
Source: Front Aging Neurosci. 2022 Aug 31;14:965190. doi: 10.3389/fnagi.2022.965190 (PMC9472554; doi:10.3389/fnagi.2022.965190)
Supplement: Supplementary file 1 [file Table_1.pdf]

Table 1. Examples of exerkines that mediate the neurological effect of physical exercise					
**Exerkine**	**Type of molecule**	**Main tissue of origin**	**Main neurological action**	**Clinical trials**	**References**
IL-6	cytokine	skeletal muscle	Modulates neuroinflammation	NCT05040321	62
Cathepsin B	cysteine protease	skeletal muscle	Increases CNS BDNF and doublecortin, proteins involved in hippocampal neurogenesis and neuronal migration	NCT01315639	85; 86
3-Hydroxybutyrate	ketone body	liver	Activates the BDNF gene promoter IV in cerebral cortical neurons	NCT03690193; NCT04466735; NCT05028114	90; 91
Lactate	alpha-hydroxy acid	skeletal muscle	Promotes BDNF expression in the hippocampus by inducing the activation of Sirtuin1 deacetylase (SIRT1)	NCT05207397; NCT04299308; NCT03999879	91; 97
BDNF	neurotrophin	brain	Regulates neuronal differentiation and survival, synaptic integrity, brain plasticity and memory function	NCT05040217; NCT02968875	91; 98
FNDC5/irisin	myokine	skeletal muscle	Induces CNS BDNF gene expression	NCT03876314	104
